# Uræmia

**Published:** 1898-08-13

**Authors:** 


### URÆMIA.

An admirable clinical lecture on uræmia, lately delivered by Sir Thomas Grainger Stewart, and published in the *Practitioner*, puts the main facts concerning that condition in a very clear light, at least so far as they appeal to the physician in his daily clinical work. We must first know what we mean by uræmia. The term is used in two senses. It is, on the one hand, regarded as being that condition of the nervous system which is apt to arise in the course of Bright's disease or other serious disease of the kidney; but a more accurate and satisfactory conception of uræmia is that which, in addition to the changes in the nervous system, recognises changes elsewhere, and especially in the alimentary system. Going back to causes, however, it is to be regarded as a result of a poisoning of the system in consequence of damage of the kidney. The symptoms, then, of uræmia have to do with the nervous system and the digestive system, and, both in regard to treatment and prognosis, especially to prognosis, it is important to bear in mind the distinction between the acute and the chronic forms of the disease, the former being often curable, the latter hardly ever. So far as the nervous system is concerned, in some cases sensory changes may be the most marked; in others, motor changes; in others, again, cerebral or mental disturbances; and cases also occur in which the organic reflexes are interfered with, as, for example, the process of respiration. Take an ordinary convulsive attack of uræmia. A patient is taken with general convulsions, with more or less profound unconsciousness. This may occur in the case of a child suffering from dropsy after scarlet fever, but it may also occur in the case of a man who, although rather out of health, perhaps, has been going about with nothing definite enough the matter with him to take him to a doctor, when he is suddenly seized with what seems to be an attack of epilepsy, after which he may be found to remain unconscious. The high tension, or renal pulse, however, soon tells us what is the origin of the attack. In both these cases the onset of the convulsion is sudden. But in the chronic form the signs of uræmic poisoning come on gradually. A man becomes somewhat torpid. "He begins to be indifferent in his manner and slow in his replies, and somewhat dragging in speech, and as you watch him day by day you find him getting gradually more torpid and more indifferent. Then you see that his tongue has become greatly furred, often of a fawn colour, like the old-fashioned drab great coat-brown and dirty. Along with that there is loss of appetite, and the man has occasionally severe fits of vomiting. He brings up mucus and partially digested food and other matter in considerable quantity. And then you further find on inquiry that he complains, if he can be got to complain at all, that he has some sort of uneasiness about his throat; and when you look into his throat you see that it is red and congested, and that, perhaps, there is a good deal of mucus clinging about the pillars of fauces, covering the tonsils and the back of the pharynx. Then you find that the patient gets worse day by day. Each day as you visit him you find that he has got more torpid and more indifferent. Later he becomes unconscious altogether," twitchings of the muscles, and perhaps actual convulsions occur, and after ten days or so, as a rule, he dies. Such chronic cases usually go on to a fatal result.

But uræmia takes many other forms according to the part of the nervous system which, in the particular individual, has a selective affinity for the particular sort of poison which is present. There may be uræmic blindness, not connected with retinal changes. Things seem dark to the patient—perhaps he is actually blind as if the centres for vision were temporarily disturbed by the action of the poison by which the uræmia is produced. Then the patient may become paralytic or paretic in some part or another, or he may suffer from uræmic dyspnœa in consequence of poisoning of the respiratory centre. Then there may be vomiting, partly of cerebral and partly of gastric origin, and there may be mental symptoms of such intensity that the patient may for a time be practically insane.

Now why do these things happen? Three theories have been suggested. Some people have maintained a mechanical theory, according to which there is dropsy of the brain, and want of blood in the vessels in consequence of the serous effusion, but there are only very few cases, if, indeed, there are are truly any, which can be explained in this way. Then it has been referred to excess of urea. But it was found that urea introduced into the blood did not produce it, and that excess of urea might be found in the blood without any convulsions. Then Frerichs, of Berlin, invented the notion that the urea becoming broken up into carbonate of ammonia and water produced the condition, but the same argument applied to that also. There was no excess of carbonate of ammonia in the blood of uræmics, and carbonate of ammonia put into the blood did not produce convulsions like those of uræmia; and so that idea went to the wall. Then, lastly, it might be due to retained waste products, and in regard to this Sir Grainger Stewart says, "I think it very probable indeed that waste products of the tissues—abnormal materials produced in the tissues during the process of tissue change—are to be blamed for the occurrence of uræmic conditions." This brings us, then, to a consideration of the conditions which precipitate a uræmic attack. Except in the purely chronic cases, in which we may imagine the uræmia to be the final product of a long-continuing accumulation, the summation of a long course of poisoning, there must be something which disturbs the balance of the system and so precipitates uræmia which would not otherwise have occurred. Now what are these causes? In the case which served as a text for the clinical lecture which we are now considering, the man got an endocarditis and secondary embolic processes, and quite independently he got some abrasions of his leg which became septic; but among other common causes may be mentioned, a drinking bout, a pregnancy, an accident by which much blood is lost, even a sudden absorption of a large dropsy loading the blood with waste products. Then an acute illness, such as a lobar pneumonia, or the inflammation of a large area of skin, or an endocarditis may suffice to precipitate a uræmia which would not otherwise have occurred—that is to say, as a consequence of the kidney disease operating alone. This is the central point which Sir Grainger Stewart wishes to emphasise, that the sudden onset is due to the occurrence of some precipitating cause, something beyond and additional to the kidney disease itself. As regards treatment, clearly what we can do is to endeavour to increase, or to find a substitute for, that elimination which in these cases is deficient—at any rate, deficient for the purposes required—and sometimes it may be necessary, especially in urgent cases of puerperal eclampsia, to bleed from the arm. But the latter cannot be often, for by the time such a course has become necessary the circulation has already become feeble and the heart is undergoing degenerative changes.

